# Supplementary material for: TFPI from erythroblasts drives heme production in central macrophages promoting erythropoiesis in polycythemia
Source: Nat Commun. 2024 May 10;15:3976. doi: 10.1038/s41467-024-48328-8 (PMC11087540; doi:10.1038/s41467-024-48328-8)
Supplement: Supplementary file 1 — Supplementary Information [file 41467_2024_48328_MOESM1_ESM.pdf]

Supplemental Figures

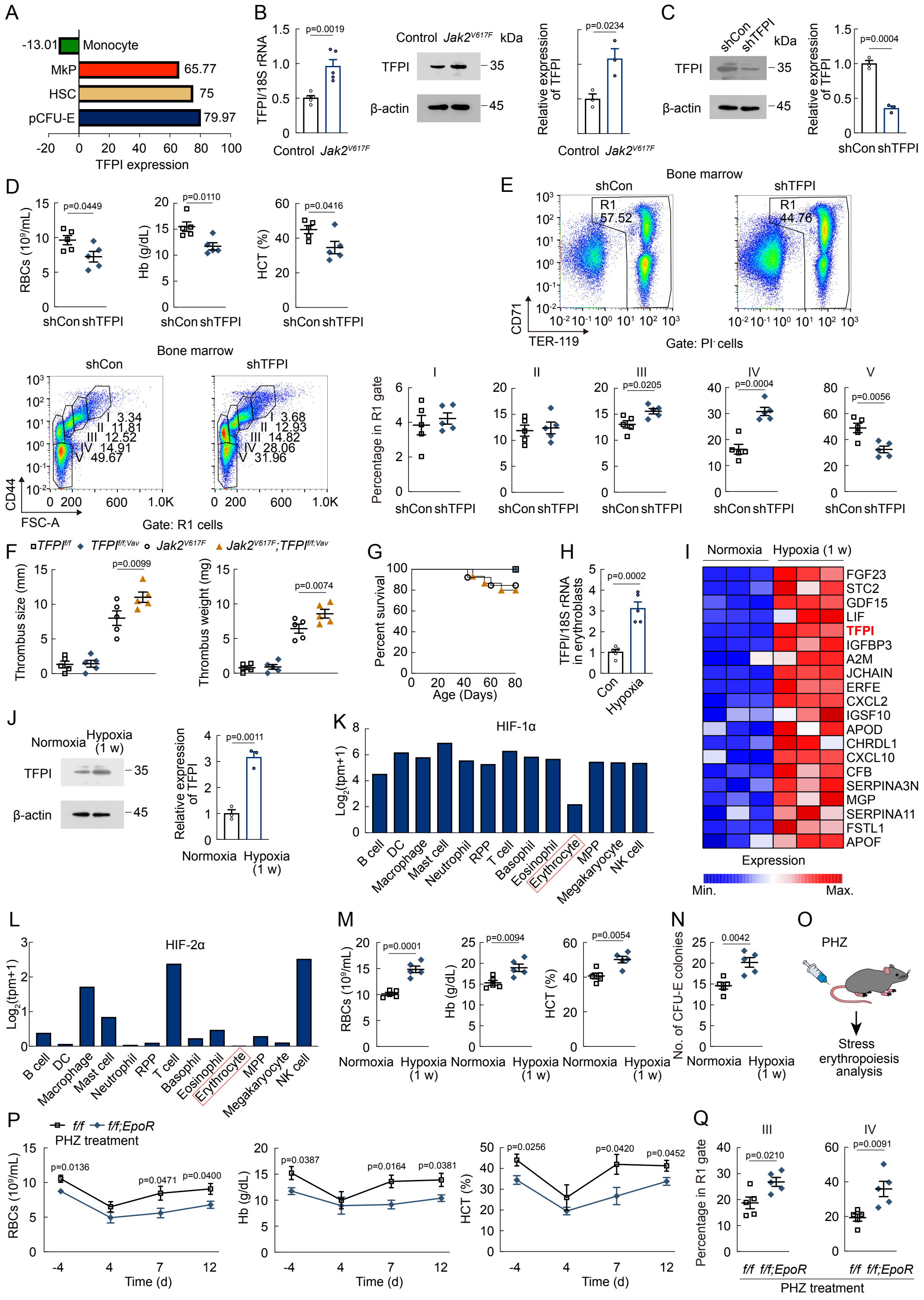

Figure S1 TFPI affects erythropoiesis in mice.

(A) Expression of TFPI mRNA in mouse BM cells. Data from Gene Expression Commons. Numbers next to bars indicate expression level. (B) TFPI expression in

Ter119$^+$ cells of *Jak2^{V617F}*-mutated mice (RT-qPCR, n = 5; Western blot, n = 3). (C) TFPI protein expression of Ter119$^+$ cells after TFPI shRNA treatment (n = 3). (D) PB RBC numbers, Hb, and HCT in shTFPI-treated mice (n = 5). (E) Frequency of erythroblast populations among BM cells in shTFPI-treated mice (n = 5). (F) Thrombus size and weight in *TFPI$^{f/f}$* mice, *Jak2^{V617F}*-mutated mice, *TFPI$^{f/f;Vav}$* mice, and *Jak2^{V617F};TFPI$^{f/f;Vav}$* mice after IVC flow restriction (n = 5). (G) Kaplan-Meier survival analysis of *TFPI$^{f/f}$* mice (n = 10), *Jak2^{V617F}*-mutated mice (n = 13), *TFPI$^{f/f;Vav}$* mice (n = 10), and *Jak2^{V617F};TFPI$^{f/f;Vav}$* mice (n = 15). (H) TFPI mRNA expression in erythroblasts of human EBI after exposed to hypoxia (n = 5). (I) Heatmap showing the top 20 up-regulated secreted protein gene expression in BM cells of hypoxia-exposed mice. (J) TFPI expression in Ter119$^+$ cells of hypoxia-exposed mice (n = 5). (K) HIF-1α mRNA levels of different cell lineages in BM based on Haemosphere (https://www.haemosphere.org). (L) HIF-2α mRNA levels of different cell lineages in BM based on Haemosphere. (M) PB RBC numbers, Hb, and HCT in hypoxia-exposed mice (n = 5). (N) CFU-E number in hypoxia-exposed mice (n = 5). (O) Protocol used for PHZ treatment. (P) PB RBC numbers, Hb and HCT in *TFPI$^{f/f;EpoR}$* mice undergoing PHZ-induced stress erythropoiesis (n = 5). (Q) Frequency of RIII and RIV erythroblast populations in *TFPI$^{f/f;EpoR}$* mice 6 d after PHZ treatment (n = 5). Statistical analysis was performed using one-way ANOVA (B-F, H, J, M-N, and P-Q). Data are shown as mean ± SEM and are representative of two (D-G and M-Q) or three (B, C, and J) independent experiments. Source data are provided as a Source Data file.

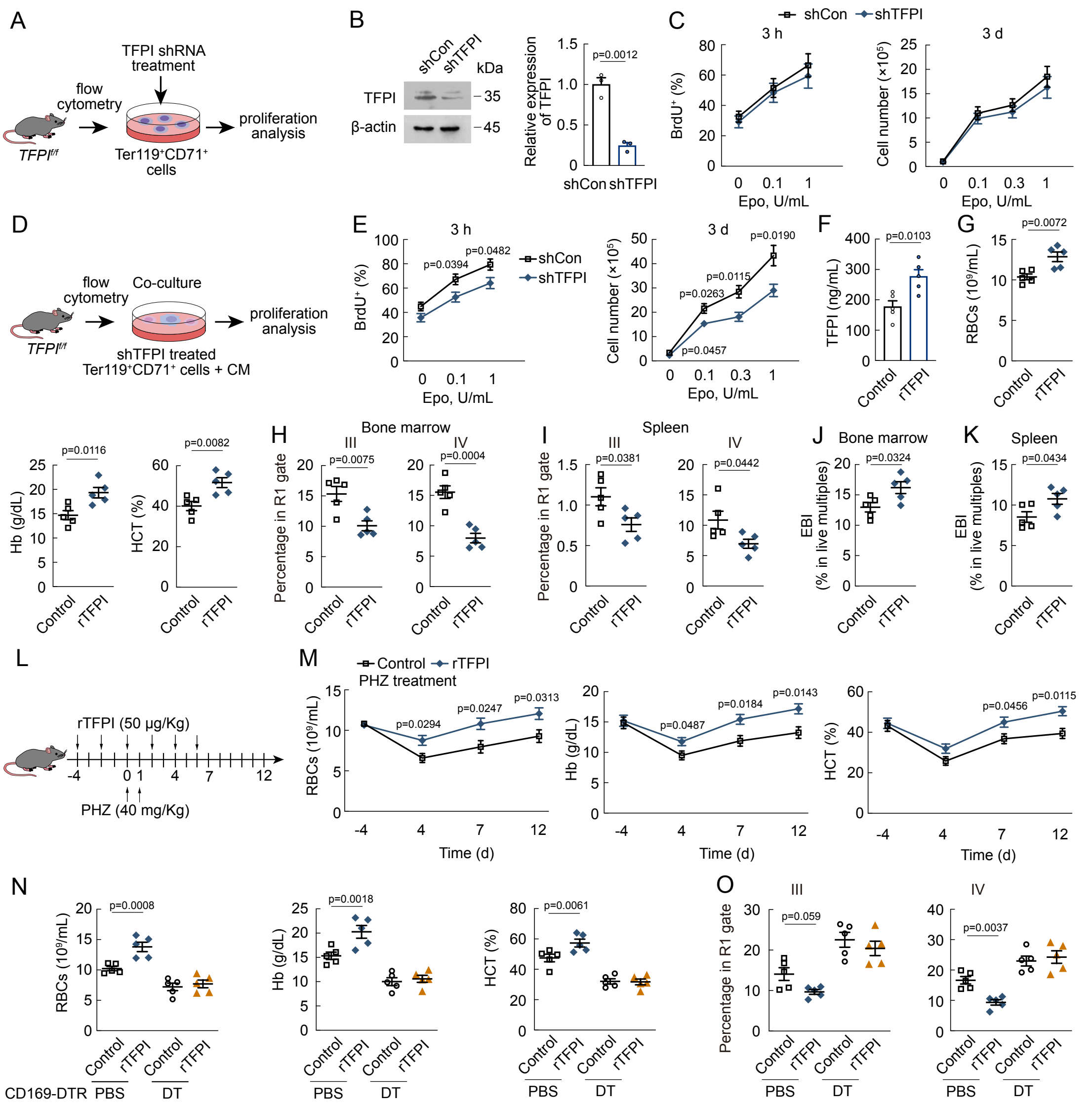

Figure S2 TFPI treatment improves erythropoiesis.

(A) Schematic diagram of *in vitro* cultivation and proliferation of Ter119$^+$CD71$^+$ cells. (B) TFPI protein expression of Ter119$^+$CD71$^+$ cells after TFPI shRNA treatment (n = 3). (C) The expansion of Ter119$^+$CD71$^+$ cells after TFPI shRNA treatment (n = 5). (D) Schematic diagram of proliferation of Ter119$^+$CD71$^+$ cells during co-culture with F4/80$^+$CD169$^+$Vcam-1$^+$ macrophages *in vitro*. (E) The expansion of Ter119$^+$CD71$^+$ cells treated with TFPI shRNA during co-culture with F4/80$^+$CD169$^+$Vcam-1$^+$ macrophages (n = 5). (F) The plasma TFPI concentration of rTFPI-treated mice (n = 5). (G) PB RBC numbers, Hb, and HCT in rTFPI-treated mice (n = 5). (H) Frequency of RIII and RIV erythroblast populations of BM in rTFPI-treated mice (n = 5). (I)

Frequency of RIII and RIV erythroblast populations of spleen in rTFPI-treated mice (n = 5). (J) EBI numbers in the BM of rTFPI-treated mice (n = 5). (K) EBI numbers in the spleen of rTFPI-treated mice (n = 5). (L) Experimental scheme for PHZ-induced stress erythropoiesis. (M) PB RBC numbers, Hb, and HCT in rTFPI-treated mice undergoing PHZ-induced stress erythropoiesis (n = 5). (N) PB RBC numbers, Hb, and HCT in CD169$^{DTR/+}$ mice after DT and rTFPI treatment (n = 5). (O) Frequency of RIII and RIV erythroblast populations of BM in CD169$^{DTR/+}$ mice after DT and rTFPI treatment (n = 5). Statistical analysis was performed using one-way ANOVA (B, E-K, and M-O). Data are shown as mean ± SEM and are representative of two (F-K and M-O) or three (B, C, and E) independent experiments. Source data are provided as a Source Data file.

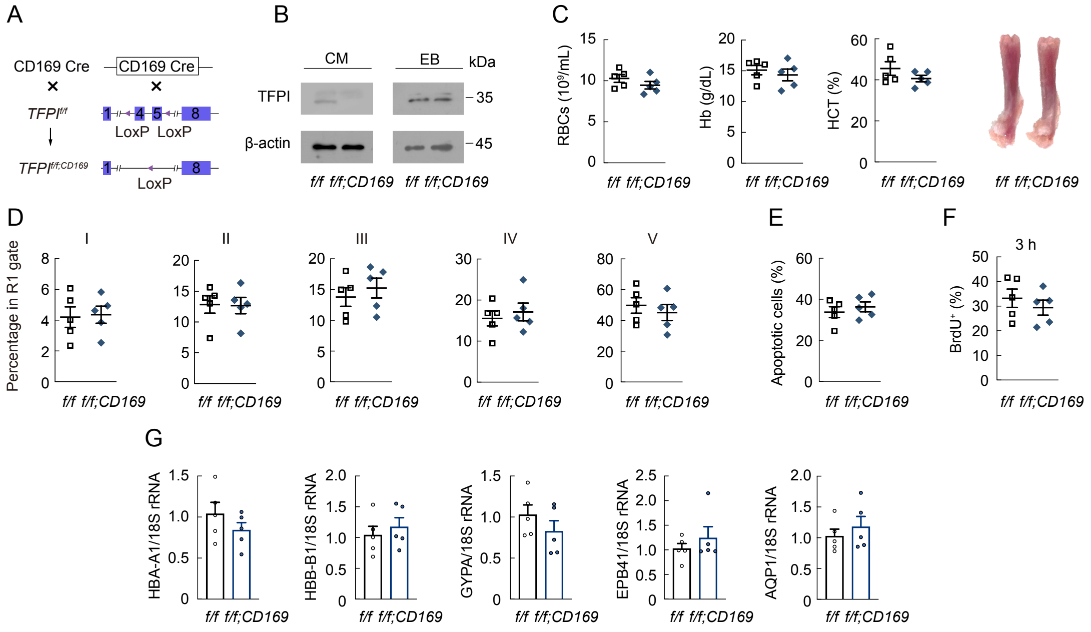

Figure S3 Macrophage-specific TFPI knockout does not affect erythropoiesis in mice.

(A) Protocol used to prepare *TFPI$^{f/f;CD169}$* mice. (B) TFPI protein expression in CM and erythroblasts of *TFPI$^{f/f;CD169}$* mice (n = 3). (C) PB RBC numbers, Hb, and HCT in *TFPI$^{f/f;CD169}$* mice (n = 5). (D) Frequency of erythroblast populations among BM cells in *TFPI$^{f/f;CD169}$* mice (n = 5). (E) Frequency of apoptotic erythroblasts among BM cells in *TFPI$^{f/f;CD169}$* mice (n = 5). (F) BrdU incorporation assay for erythroblasts proliferation of *TFPI$^{f/f;CD169}$* mice (n = 5). (G) HBA-A1, HBB-B1, GYPA, EPB41 and AQP1 mRNA expression in erythroblasts among BM cells of *TFPI$^{f/f;CD169}$* mice (n = 5). Data are shown as mean ± SEM and are representative of two (C and D) or three (B, E-G) independent experiments. Source data are provided as a Source Data file.

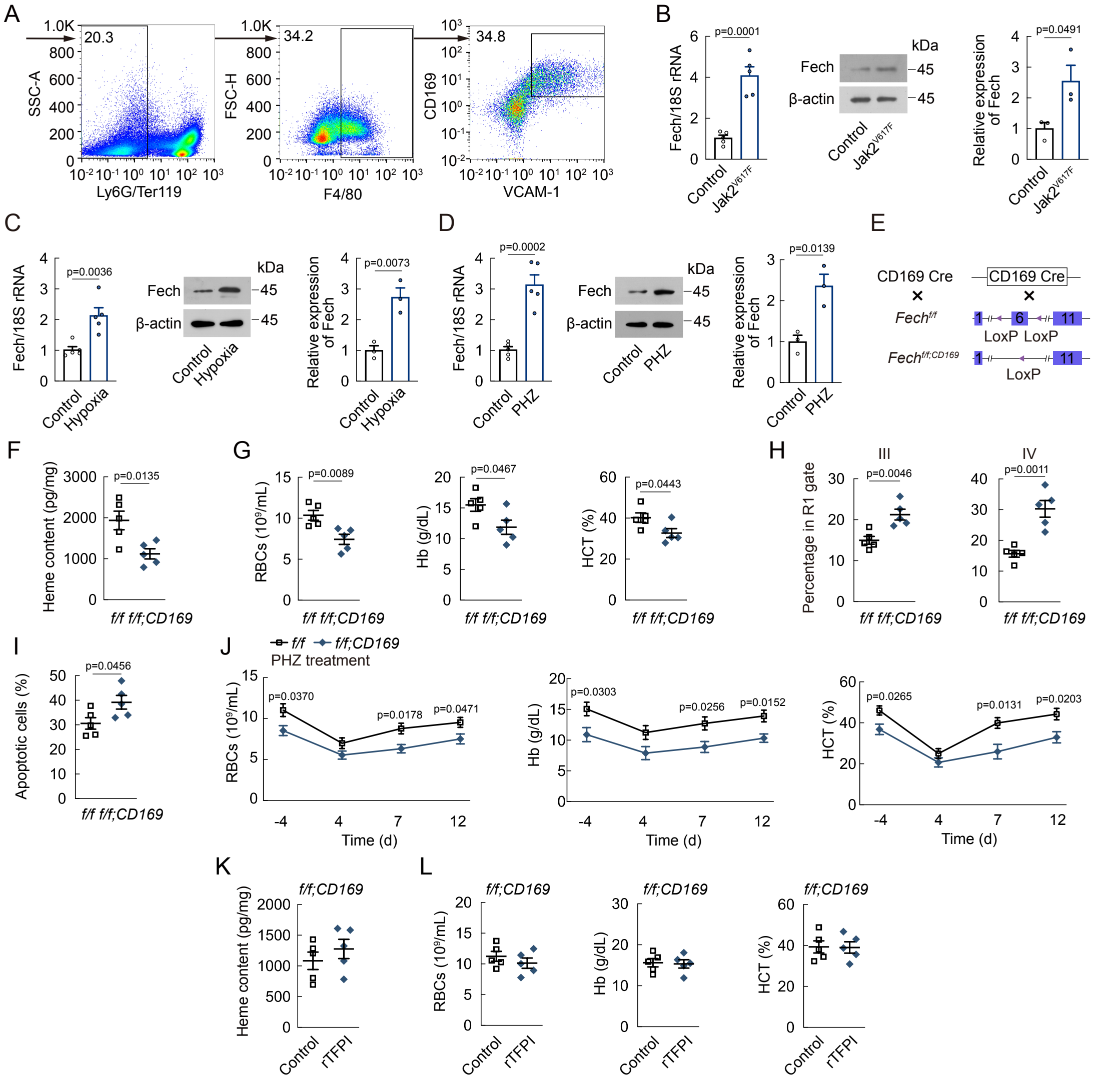

Figure S4 Heme in F4/80$^+$CD169$^+$Vcam-1$^+$ macrophages improves erythropoiesis. (A) Representative FACS plots of the F4/80$^+$CD169$^+$Vcam-1$^+$ macrophage gating strategy. (B) Fech expression in F4/80$^+$CD169$^+$Vcam-1$^+$ macrophages of *Jak2*^{V617F}-mutated mice (RT-qPCR, n = 5; Western blot, n = 3). (C) Fech expression in F4/80$^+$CD169$^+$Vcam-1$^+$ macrophages of hypoxia-exposed mice (RT-qPCR, n = 5; Western blot, n = 3). (D) Fech expression in F4/80$^+$CD169$^+$Vcam-1$^+$ macrophages of PHZ-treated mice (RT-qPCR, n = 5; Western blot, n = 3). (E) Protocol to prepare *Fech*$^{f/f;CD169}$ mice. (F) Heme content in F4/80$^+$CD169$^+$Vcam-1$^+$ macrophages of *Fech*$^{f/f;CD169}$ mice (n = 5). (G) PB RBC numbers, Hb, and HCT in *Fech*$^{f/f;CD169}$ mice (n = 5). (H) Frequency of RIII and RIV erythroblast populations in *Fech*$^{f/f;CD169}$ mice (n =

5). (I) Frequency of apoptotic erythroblasts among BM cells in *Fech*$^{f/f;CD169}$ mice (n = 5). (J) PB RBC numbers, Hb, and HCT in *Fech*$^{f/f;CD169}$ mice after PHZ treatment (n = 5). (K) Heme content in F4/80^{+}CD169^{+}Vcam-1^{+} macrophages of *Fech*$^{f/f;CD169}$ mice after rTFPI treatment (n = 5). (L) PB RBC numbers, Hb, and HCT in *Fech*$^{f/f;CD169}$ mice after rTFPI treatment (n = 5). Statistical analysis was performed using one-way ANOVA (B-D, and F-L). Data are shown as mean ± SEM and are representative of two (F-L) or three (B-D) independent experiments. Source data are provided as a Source Data file.

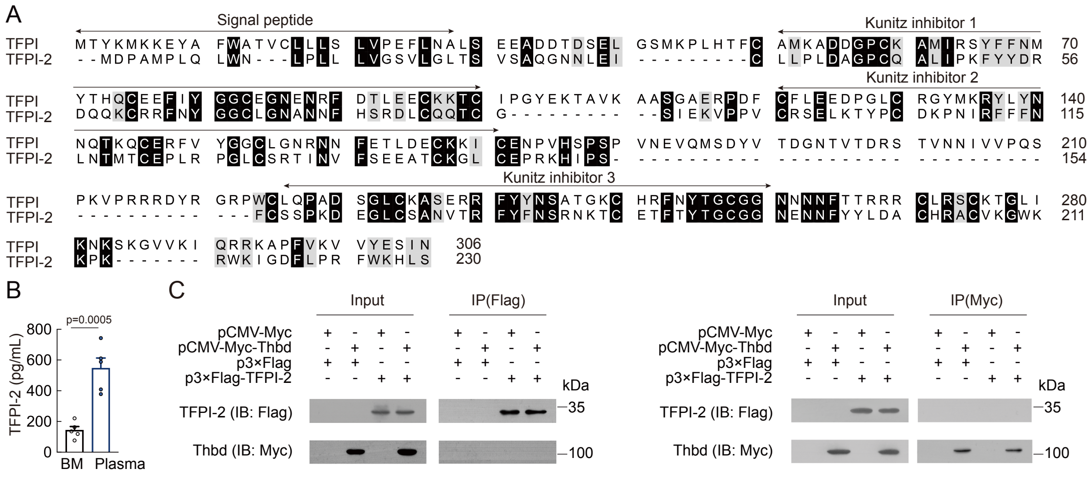

Figure S5 Thbd does not interact with TFPI-2.

(A) Multiple alignment of the amino acid sequences of TFPI and TFPI-2 in mice. (B) TFPI-2 protein expression in BM and plasma (n = 5). (C) Co-IP analysis of the interaction between Thbd and TFPI-2 in HEK293T cells (n = 2). Statistical analysis was performed using one-way ANOVA (B). Data are shown as mean ± SEM and are representative of two (C) or three (B) independent experiments. Source data are provided as a Source Data file.

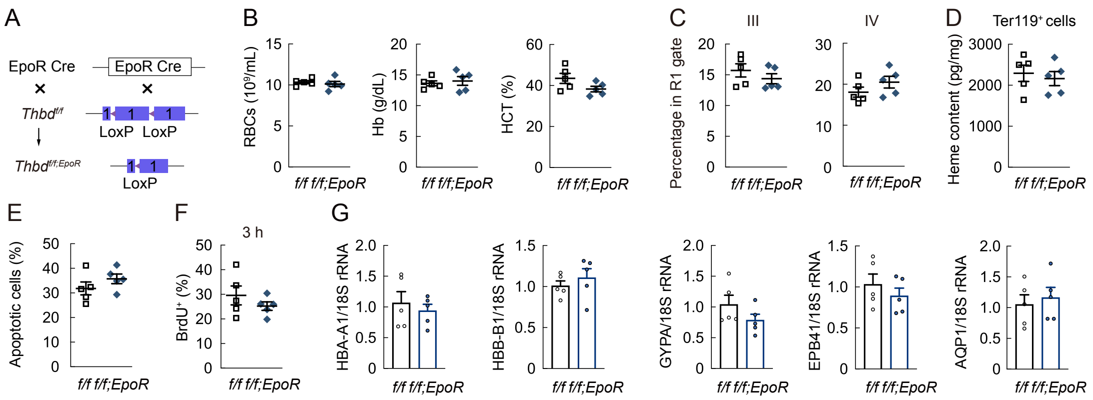

Figure S6 Erythroid-specific Thbd knockout does not affect erythropoiesis in mice.

(A) Protocol used to prepare *Thbd*$^{f/f;EpoR}$ mice. (B) PB RBC numbers, Hb, and HCT in *Thbd*$^{f/f;EpoR}$ mice (n = 5). (C) Frequency of erythroblast populations among BM cells in *Thbd*$^{f/f;EpoR}$ mice (n = 5). (D) Heme content in Ter119^{+} cells of *Thbd*$^{f/f;EpoR}$ mice (n = 5). (E) Frequency of apoptotic erythroblasts among BM cells in *Thbd*$^{f/f;EpoR}$ mice (n = 5). (F) BrdU incorporation assay for erythroblasts proliferation of *Thbd*$^{f/f;EpoR}$ mice (n = 5). (G) HBA-A1, HBB-B1, GYPA, EPB41 and AQP1 mRNA expression in erythroblasts among BM cells of *Thbd*$^{f/f;EpoR}$ mice (n = 5). Data are shown as mean ± SEM and are representative of two (B-D) or three (E-G) independent experiments. Source data are provided as a Source Data file.

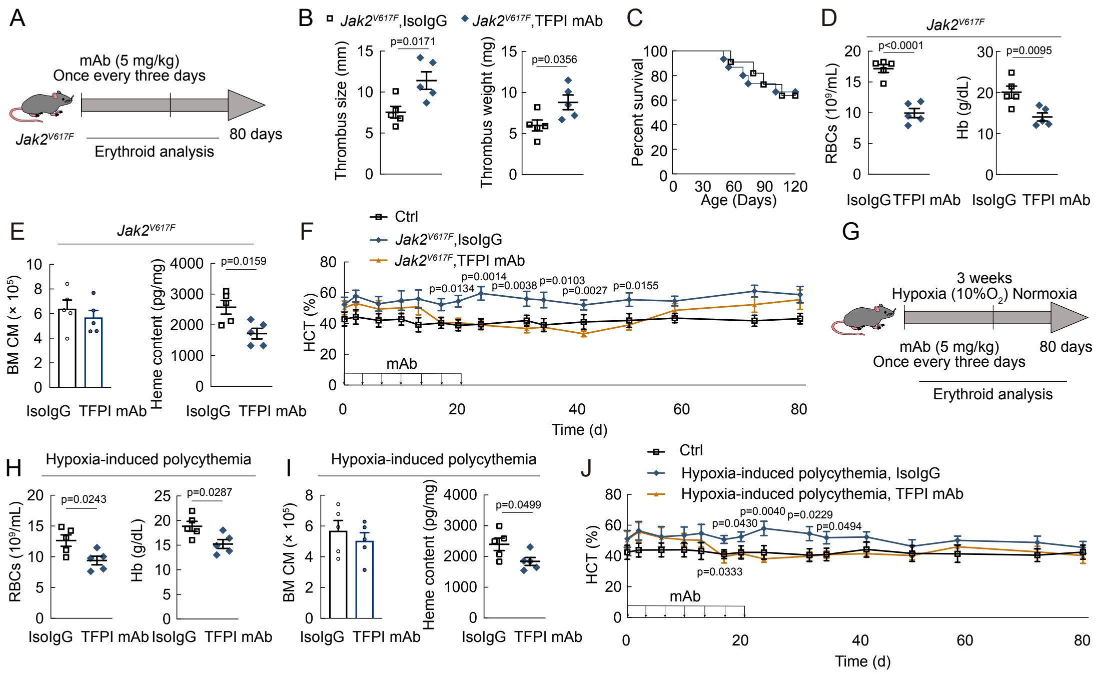

Figure S7 Effects of anti-TFPI antibody on hypoxia- and *Jak2^{V617F}*-induced polycythemia.

(A) Schematic of experimental design for TFPI mAb treatment of *Jak2^{V617F}*-mutated mice. (B) Thrombus size and weight in *Jak2^{V617F}*-mutated mice and TFPI mAb-treated *Jak2^{V617F}*-mutated mice after IVC flow restriction. (C) Kaplan-Meier survival analysis of *Jak2^{V617F}*-mutated mice (n = 11) and TFPI mAb-treated *Jak2^{V617F}*-mutated mice (n = 15). (D) PB RBC numbers and Hb in *Jak2^{V617F}*-mutated mice after TFPI mAb treatment (n = 5). (E) The numbers of F4/80^{+}CD169^{+}Vcam-1^{+} macrophages per femur and F4/80^{+}CD169^{+}Vcam-1^{+} macrophage heme content of *Jak2^{V617F}*-mutated mice after TFPI mAb treatment (n = 5). (F) HCT in control and *Jak2^{V617F}*-mutated mice after treated with IsoIgG and TFPI mAb (n = 5). (G) Schematic of experimental design for construction of a mouse model of hypoxia-induced polycythemia. (H) PB RBC numbers and Hb in hypoxia-induced polycythemia mice after TFPI mAb treatment for 3 weeks (n = 5). (I) The numbers of F4/80^{+}CD169^{+}Vcam-1^{+} macrophages per femur and F4/80^{+}CD169^{+}Vcam-1^{+} macrophage heme content of hypoxia-induced

polycythemia mice after TFPI mAb treatment for 3 weeks (n = 5). (J) HCT in control and hypoxia-induced polycythemia mice after treated with IsoIgG and TFPI mAb (n = 5). Statistical analysis was performed using one-way ANOVA (B,D-F, and H-J). Data are shown as mean ± SEM and are representative of two independent experiments. Source data are provided as a Source Data file.

Supplemental Tables

Supplementary Table 1. The list of candidate TFPI-interacting proteins identified by yeast-two-hybrid analyses.

Ensembl	Positive gene identified	positive clones/total identical clones
ENSMUSG00000031444	F10	3/30
ENSMUSG00000074743	Thbd	8/30
ENSMUSG00000055436	Srsf11	15/30
ENSMUSG00000027249	F2	1/30
ENSMUSG00000024097	Srsf7	17/30
ENSMUSG00000022961	Son	18/30

Supplementary Table 2. Characteristics of patient and healthy cohorts.

	healthy cohorts	patient cohorts
Number of patient/healthy cohort	18	21
Male	7	10
Female	11	11
Median age for samples (years)	40	37

Supplementary Table 3. Primers used for RT-qPCR.

**RT-qPCR**	
Genes	Primers
Mouse	
TFPI	Forword 5'CCTTCCCCAGTGAATGAGGT3'
	Reverse 5'ATCCACTGTCTGCTGGTTGA3'
Fech	Forword 5'TGACCACATTGAGACGCTCT3'
	Reverse 5'GCTTGTTGGACTGGATGTGG3'
Thbd	Forword 5'GGCTGGAGATGACCCAGATA3'
	Reverse 5'GAAACAACGGTAAGGGCAAA3'
CD71	Forword 5'AGCCAGATCAGCATTCTCTAACT3'
	Reverse 5'GCCTTCATGTTATTGTCGGCAT3'
ALAS2	Forword 5'CACCTATGCTTAAGGAGCCA3'

	Reverse 5'CAGAAGCACACAGGAAAGCA3'
ALAD	Forword 5'GTTCTGCACAGCGGCTACTT3'
	Reverse 5'AGCTGGTTTACGCCATACCTG3'
HMBS	Forword 5'AAGGGCTTTTCTGAGGCACC3'
	Reverse 5'AGTTGCCCATCTTTCATCACTG3'
UROS	Forword 5'GCAGTGAAGCTGTGTTTGGA3'
	Reverse 5'GCAAGCTTTTCTGCATTTCC3'
UROD	Forword 5'CCTCTGGATGCTGCCATAAT3'
	Reverse 5'TAAGGGTGATGGCTTGGAAC3'
CPOX	Forword 5'ATGCCCTTCTTGGGAACTTT3'
	Reverse 5'TAGCCCTGGCTGGACTAGAA3'
PC	Forword 5'GGTGCTCATCCACACTTCCT3'
	Reverse 5'GCAGATGGGCACTATGGTTT3'
TF	Forword 5'TGCTTCTCGACCACAGACAC3'
	Reverse 5'TAAAAACTTTGGGGCGTTTG3'
Human	
TFPI	Forword 5'ATGGAACCCAGCTCAATGCT3'
	Reverse 5'GGCACGACACAATCCTCTGT3'
Thbd	Forword 5'ACATCCTGGACGACGGTTTC3'
	Reverse 5'CGCAGATGCACTCGAAGGTA3'
TF	Forword 5'AGACAGCCCGGTAGAGTGTA3'
	Reverse 5'AGCCAGGATGATGACAAGGAT3'
Fech	Forword 5'TTGTTCTCTAAGGCCCTGGC3'
	Reverse 5'GCGGACAGCTCAGGGTCA3'

Supplementary Table 4. Target sequences for shRNA.

**shRNA**	
Lentivirus	Sequences
Mouse TFPI shRNA	5'CCATGCAAAGCAATGATAAGGAGTT3'
Human TFPI shRNA	5'ACAGTGTGAACGTTTCAAGTA3'
Mouse GATA1 shRNA	5'GAGGAAATGGAGGAGGCCTTGAAGA3'
Mouse Thbd shRNA	5'GCCCACATCTCTAGTACCTACAACA3'
Mouse aPC shRNA	5'CATGCGCTGCAAGTCCACTGTGAAT3'
