# Supplementary material for: TFPI from erythroblasts drives heme production in central macrophages promoting erythropoiesis in polycythemia
Source: Nat Commun. 2024 May 10;15:3976. doi: 10.1038/s41467-024-48328-8 (PMC11087540; doi:10.1038/s41467-024-48328-8)
Supplement: Supplementary file 3 — Reporting Summary [file 41467_2024_48328_MOESM3_ESM.pdf]

nature portfolio

Corresponding author(s): Xin-Jiang Lu

Last updated by author(s): Mar 8, 2024

# Reporting Summary

Nature Portfolio wishes to improve the reproducibility of the work that we publish. This form provides structure for consistency and transparency in reporting. For further information on Nature Portfolio policies, see our Editorial Policies and the Editorial Policy Checklist.

## Statistics

For all statistical analyses, confirm that the following items are present in the figure legend, table legend, main text, or Methods section.

| n/a | Confirmed | |
|---|---|---|
| ☐ | ☒ | The exact sample size (*n*) for each experimental group/condition, given as a discrete number and unit of measurement |
| ☐ | ☒ | A statement on whether measurements were taken from distinct samples or whether the same sample was measured repeatedly |
| ☐ | ☒ | The statistical test(s) used AND whether they are one- or two-sided<br>*Only common tests should be described solely by name; describe more complex techniques in the Methods section.* |
| ☒ | ☐ | A description of all covariates tested |
| ☐ | ☒ | A description of any assumptions or corrections, such as tests of normality and adjustment for multiple comparisons |
| ☐ | ☒ | A full description of the statistical parameters including central tendency (e.g. means) or other basic estimates (e.g. regression coefficient) AND variation (e.g. standard deviation) or associated estimates of uncertainty (e.g. confidence intervals) |
| ☐ | ☒ | For null hypothesis testing, the test statistic (e.g. *F*, *t*, *r*) with confidence intervals, effect sizes, degrees of freedom and *P* value noted<br>*Give P values as exact values whenever suitable.* |
| ☒ | ☐ | For Bayesian analysis, information on the choice of priors and Markov chain Monte Carlo settings |
| ☒ | ☐ | For hierarchical and complex designs, identification of the appropriate level for tests and full reporting of outcomes |
| ☒ | ☐ | Estimates of effect sizes (e.g. Cohen's *d*, Pearson's *r*), indicating how they were calculated |

*Our web collection on statistics for biologists contains articles on many of the points above.*

## Software and code

Policy information about availability of computer code

| | |
|---|---|
| Data collection | Flow cytometric data was collected by Moflo Astrios EQ and BD Fortessa.<br>RT-qPCR data was collected by CFX-Touch 96<br>Complete blood count was collected by XN-1000V<br>cytospin images were collected by Olympus BX61<br>RNA sequencing data was collected by Illumina Novaseq 6000 |
| Data analysis | flow cytometry data was analyzed with FlowJo 10.0.7<br>Western blot quantification were analyzed by ImageJ 1.53<br>All statistical data analysis was performed using Graphpad Prism 9 and IBM SPSS Statistics 23<br>RNA sequencing data was analyzed by TBtools 2.019, DESeq2 R packages 1.38.3, ggplot2 R packages 3.4.2, DAVID (https://david.ncifcrf.gov/) |

For manuscripts utilizing custom algorithms or software that are central to the research but not yet described in published literature, software must be made available to editors and reviewers. We strongly encourage code deposition in a community repository (e.g. GitHub). See the Nature Portfolio guidelines for submitting code & software for further information.

# Data

Policy information about availability of data

All manuscripts must include a data availability statement. This statement should provide the following information, where applicable:

- Accession codes, unique identifiers, or web links for publicly available datasets
- A description of any restrictions on data availability
- For clinical datasets or third party data, please ensure that the statement adheres to our policy

The RNA-seq data under this study are available at GEO under accession code GSE224993 [https://www.ncbi.nlm.nih.gov/geo/query/acc.cgi?acc=GSE224993] and GSE224994 [https://www.ncbi.nlm.nih.gov/geo/query/acc.cgi?acc=GSE224994]. The data generated in this study are provided in the Source Data file. Source data are provided with this paper.

# Research involving human participants, their data, or biological material

Policy information about studies with human participants or human data. See also policy information about sex, gender (identity/presentation), and sexual orientation and race, ethnicity and racism.

| | |
|---|---|
| Reporting on sex and gender | Sex or gender was not considered in the study design. |
| Reporting on race, ethnicity, or other socially relevant groupings | Race, ethnicity, or other socially relevant groupings were not considered. |
| Population characteristics | Totally 39 participants from the Second Affiliated Hospital of Zhejiang University School of Medicine were recruited in our study, consisting of 18 healthy donors (20-68 years old, mean = 40; 11 females and 7 males), and 21 JAK2V617F-mutated patients (16–65 years old, mean = 37; 11 females and 10 males). |
| Recruitment | In our study, we recruited a total of 39 participants, including healthy donors and JAK2V617F-mutated patients. Informed consent was obtained from all subjects. Human blood samples were collected following informed consent at the Second Affiliated Hospital of Zhejiang University School of Medicine without monetary compensation. No self-selection bias or other biases have been recorded. |
| Ethics oversight | JAK2V617F-mutated samples and healthy donor samples were obtained from the Second Affiliated Hospital of Zhejiang University School of Medicine. Informed consent was obtained from all subjects. The study was approved by the Ethics Committee of Second Affiliated Hospital of Zhejiang University (No. 20230705). |

Note that full information on the approval of the study protocol must also be provided in the manuscript.

# Field-specific reporting

Please select the one below that is the best fit for your research. If you are not sure, read the appropriate sections before making your selection.

☒ Life sciences ☐ Behavioural & social sciences ☐ Ecological, evolutionary & environmental sciences

For a reference copy of the document with all sections, see nature.com/documents/nr-reporting-summary-flat.pdf

# Life sciences study design

All studies must disclose on these points even when the disclosure is negative.

| | |
|---|---|
| Sample size | No statistical method was used to predetermine the sample size. For animal experiments, sample size was chosen based on preliminary data and observed effect sizes. More than 5 mice were used per group. For human study, 18 healthy donors and 21 JAK2V617F-mutated patients were recruited (PMID: 38097578 and PMID: 36494342). For in vitro experiements, sample size for each experiments have indicated in figure legend, protein interaction-interaction experiments were repeated two times (PMID: 38097609 and PMID: 38448435), and other experiments were repeated at least three times. |
| Data exclusions | No data were excluded. |
| Replication | The reproducibility for each analysis confirmed at least two times. Number of repeats is provided in the figure legends. |
| Randomization | Animals were randomly divided into experimental and control groups. Cells grown under the same conditions randomly allocated into different groups without bias, and wells were randomly selected for different treatments. |
| Blinding | For animal experiments, the investigators could not be blinded to sample allocations because mouse genotyping, treatment and end-point analysis were preformed. For in vitro experiments, the investigators were not blinded during data processing and analysis of in vitro data. For data analysis, if feasible, the investigators were blinded to the experimental conditions when assessing the outcomes. |

# Reporting for specific materials, systems and methods

We require information from authors about some types of materials, experimental systems and methods used in many studies. Here, indicate whether each material, system or method listed is relevant to your study. If you are not sure if a list item applies to your research, read the appropriate section before selecting a response.

## Materials & experimental systems

| n/a | Involved in the study |
|---|---|
| ☐ | ☒ Antibodies |
| ☐ | ☒ Eukaryotic cell lines |
| ☒ | ☐ Palaeontology and archaeology |
| ☐ | ☒ Animals and other organisms |
| ☒ | ☐ Clinical data |
| ☒ | ☐ Dual use research of concern |
| ☒ | ☐ Plants |

## Methods

| n/a | Involved in the study |
|---|---|
| ☒ | ☐ ChIP-seq |
| ☐ | ☒ Flow cytometry |
| ☒ | ☐ MRI-based neuroimaging |

## Antibodies

Antibodies used

Anti-mouse TFPI antibody (1:500, ab180619) Abcam
Anti-human TFPI antibody (1:500, ab260042) Abcam
Anti-mouse Thbd antibody (1:1000, ab230010) Abcam
Anti-human Thbd antibody (1:1000, ab109189) Abcam
Anti-mouse PC antibody (1:1000, ab313386) Abcam
Anti-mouse Fech antibody (1:1000, 14466-1-AP) Proteintech
Anti-mouse GATA1 antibody (1:1000, sc-265) Santa Cruz Biotechnology
Anti-Flag antibody (1:1000, F3165) Sigma-Aldrich
Anti-Myc antibody (1:1000, 2276) Cell Signaling Technology
Anti-GST antibody (1:1000, 2622) Cell Signaling Technology
Anti-p-GATA1 antibody (1:500, PA5-104243) Thermo Fisher Scientific
Anti-β-actin antibody (1:1000, sc-47778) Santa Cruz Biotechnology
Anti-GAPDH antibody (1:1000, sc-365062) Santa Cruz Biotechnology
Goat Anti-rabbit IgG H&L (HRP) (1:2000, ab6721) Abcam
Goat Anti-mouse IgG H&L (HRP) (1:2000, ab205719) Abcam
Anti-TFPI Recombinant Antibody (Concizumab) (TAB-734) Creative Biolabs
PE-anti-mouse TER-119/Erythroid cells (1:100, 116207) Biolegend
PE/Cyanine7-anti-mouse CD71 (1:100, 113811) Biolegend
APC-anti-mouse/human CD44 (1:100, 103011) Biolegend
PE-anti-mouse Ly6G (1:100, 127608) Biolegend
FITC-anti-mouse TER-119/Erythroid cells (1:100, 116205) Biolegend
BV421-anti-mouse F4/80 (1:25, 123131) Biolegend
APC-anti-mouse c-Kit (1:50, 105811) Biolegend
Anti-mouse lineage cocktail (1:100, 133301) Biolegend
APC-anti-mouse VCAM-1 (1:100, 105717) Biolegend

Validation

All antibodies are from commercial companies and are well validated by manufacturer and widely used in researches. Their validation data are available on the manufacturer's websites:
Anti-mouse TFPI antibody (1:500, ab180619) Abcam
https://www.abcam.co.jp/products/primary-antibodies/tfpi-antibody-ab180619.html
Anti-human TFPI antibody (1:500, ab260042) Abcam
https://www.abcam.co.jp/products/primary-antibodies/tfpi-antibody-epr22977-133-ab260042.html
Anti-mouse Thbd antibody (1:1000, ab230010) Abcam
https://www.abcam.co.jp/products/primary-antibodies/thrombomodulin-antibody-epr18217-209-ab230010.html
Anti-human Thbd antibody (1:1000, ab109189) Abcam
https://www.abcam.co.jp/products/primary-antibodies/thrombomodulin-antibody-epr4051-ab109189.html
Anti-mouse PC antibody (1:1000, ab313386) Abcam
https://www.abcam.co.jp/products/primary-antibodies/protein-c-antibody-epr28195-90-ab313386.html
Anti-mouse Fech antibody (1:1000, 14466-1-AP) Proteintech
https://www.ptglab.com/products/FECH-Antibody-14466-1-AP.htm
Anti-mouse GATA1 antibody (1:1000, sc-265) Santa Cruz Biotechnology
https://www.scbt.com/p/gata-1-antibody-n6?requestFrom=search
Anti-Flag antibody (1:1000, F3165) Sigma-Aldrich
https://www.sigmaaldrich.com/SG/en/search/f3165?
focus=products&page=1&perpage=30&sort=relevance&term=F3165&type=product
Anti-Myc antibody (1:1000, 2276) Cell Signaling Technology
https://www.cellsignal.com/products/primary-antibodies/myc-tag-9b11-mouse-mab/2276
Anti-GST antibody (1:1000, 2622) Cell Signaling Technology
https://www.cellsignal.com/products/primary-antibodies/gst-tag-antibody/2622
Anti-p-GATA1 antibody (1:500, PA5-104243) Thermo Fisher Scientific

https://www.thermofisher.cn/cn/zh/antibody/product/Phospho-GATA1-Ser310-Antibody-Polyclonal/PA5-104243
Anti-β-actin antibody (1:1000, sc-47778) Santa Cruz Biotechnology
https://www.scbt.com/p/beta-actin-antibody-c4?requestFrom=search
Anti-GAPDH antibody (1:1000, sc-365062) Santa Cruz Biotechnology
https://www.scbt.com/p/gapdh-antibody-g-9?search-input=
Goat Anti-rabbit IgG H&L (HRP) (1:2000, ab6721) Abcam
https://www.abcam.co.jp/products/secondary-antibodies/goat-rabbit-igg-hl-hrp-ab6721.html
Goat Anti-mouse IgG H&L (HRP) (1:2000, ab205719) Abcam
https://www.abcam.com/products/secondary-antibodies/goat-mouse-igg-hl-hrp-ab205719.html
PE-anti-mouse TER-119/Erythroid cells (1:100, 116207) Biolegend
https://www.biolegend.com/nl-be/products/pe-anti-mouse-ter-119-erythroid-cells-antibody-1867
PE/Cyanine7-anti-mouse CD71 (1:100, 113811) Biolegend
https://www.biolegend.com/nl-be/products/pe-cyanine7-anti-mouse-cd71-antibody-6185
APC-anti-mouse/human CD44 (1:100, 103011) Biolegend
https://www.biolegend.com/nl-be/products/apc-anti-mouse-human-cd44-antibody-312
PE-anti-mouse Ly6G (1:100, 127608) Biolegend
https://www.biolegend.com/nl-be/products/pe-anti-mouse-ly-6g-antibody-4777
FITC-anti-mouse TER-119/Erythroid cells (1:100, 116205) Biolegend
https://www.biolegend.com/nl-be/products/fitc-anti-mouse-ter-119-erythroid-cells-antibody-1865
BV421-anti-mouse F4/80 (1:25, 123131) Biolegend
https://www.biolegend.com/nl-be/products/brilliant-violet-421-anti-mouse-f4-80-antibody-7199
APC-anti-mouse c-Kit (1:50, 105811) Biolegend
https://www.biolegend.com/nl-be/products/apc-anti-mouse-cd117-c-kit-antibody-72
Anti-mouse lineage cocktail (1:100, 133301) Biolegend
https://www.biolegend.com/nl-be/products/fitc-anti-mouse-lineage-cocktail-with-isotype-ctrl-5803
APC-anti-mouse VCAM-1 (1:100, 105717) Biolegend
https://www.biolegend.com/nl-be/products/apc-anti-mouse-cd106-antibody-6079

## Eukaryotic cell lines

Policy information about cell lines and Sex and Gender in Research

| | |
|---|---|
| Cell line source(s) | HEK293T (ATCC, #CRL-3216) |
| Authentication | The cell line was authenticated by STR profiling. |
| Mycoplasma contamination | All cell lines were tested negative for mycoplasma contamination. |
| Commonly misidentified lines (See ICLAC register) | No commonly misidentified lines were used in our study. |

## Animals and other research organisms

Policy information about studies involving animals; ARRIVE guidelines recommended for reporting animal research, and Sex and Gender in Research

| | |
|---|---|
| Laboratory animals | C57BL/6 mice were purchased from Zhejiang Provincial Laboratory Animal Center. EpoR-Cre mice were kindly provided by Stuart H. Orkin (Harvard Medical School, Boston, MA). LysM-Cre (Stock# 004781) and Jak2V617F/+ (Stock# 031658) mice were purchased from The Jackson Laboratory. Vav-iCre (Stock# C001019) and TFPIf/f (Stock# S-CKO-06215) mice were purchased from Cyagen Biosciences Inc. (Suzhou, China). CD169-Cre (Stock# NM-KI-215032) and Thbdf/f (Stock# NM-CKO-2101896) mice were obtained from Shanghai Model Organisms Center, Inc. (Shanghai, China). All mice were housed under 12h-light-dark cycle with controlled temperature (22± 2°C) and humidity (30-70%) in a specific pathogen-free barrier facility. Experiments were performed on 6-8-week-old mice. All mice stains used in this study are described in Methods section of the manuscript. |
| Wild animals | No wild animals were used in this study. |
| Reporting on sex | Mice of both sexes were used in this study. |
| Field-collected samples | No field-collected samples were used. |
| Ethics oversight | All mice were housed in a specific pathogen-free barrier facility. The experimental conditions and procedures were approved by the Zhejiang University Institutional Animal Care and Use Committee and were consistent with the National Institutes of Health Guide for the Care and Use of Laboratory Animals. |

Note that full information on the approval of the study protocol must also be provided in the manuscript.

# Plants

Seed stocks

*Report on the source of all seed stocks or other plant material used. If applicable, state the seed stock centre and catalogue number. If plant specimens were collected from the field, describe the collection location, date and sampling procedures.*

Novel plant genotypes

*Describe the methods by which all novel plant genotypes were produced. This includes those generated by transgenic approaches, gene editing, chemical/radiation-based mutagenesis and hybridization. For transgenic lines, describe the transformation method, the number of independent lines analyzed and the generation upon which experiments were performed. For gene-edited lines, describe the editor used, the endogenous sequence targeted for editing, the targeting guide RNA sequence (if applicable) and how the editor was applied.*

Authentication

*Describe any authentication procedures for each seed stock used or novel genotype generated. Describe any experiments used to assess the effect of a mutation and, where applicable, how potential secondary effects (e.g. second site T-DNA insertions, mosiacism, off-target gene editing) were examined.*

# Flow Cytometry

## Plots

Confirm that:

☒ The axis labels state the marker and fluorochrome used (e.g. CD4-FITC).

☒ The axis scales are clearly visible. Include numbers along axes only for bottom left plot of group (a 'group' is an analysis of identical markers).

☒ All plots are contour plots with outliers or pseudocolor plots.

☒ A numerical value for number of cells or percentage (with statistics) is provided.

## Methodology

Sample preparation

BM cells were isolated by thoroughly flushing tibias, femurs, and humeri using a 5 ml polystyrene tube with a strainer (BD Biosciences). Spleens were mashed through a 70 μm nylon filter. For EBI isolation, BM was flushed gently with Iscove's modified Dulbecco's medium (IMDM) containing 3.5% sodium citrate and 20% fetal calf serum solution using an 18G syringe without centrifugation. Spleen was cut into small pieces, and incubated in RPMI1640 containing 0.075% Collagenase IV and 0.004% (m/v) DNase I for 30min. The suspension was passed through an 18-guage needle, washed and resuspended in IMDM containing 3.5% sodium citrate and 20% fetal calf serum solution.

Instrument

Cells were analysed on BD Fortessa (BD Biosciences), and were sorted with Moflo Astrios (Beckman).

Software

FlowJo (10.0.7).

Cell population abundance

At least 10000 events were acquired for cells in each assay. For FACS sorting, the sorted cells were reanalyzed to assess purity. More than 75% purity was achieved.

Gating strategy

Cells were initially gated over the 2D density of events on forward and side scatter (FSC-A/SSC-A) to exclude cell debris. Sequential gating/sorting strategies are provided in Figure 1, Figure 2, supplemental Figure 1, and supplemental Figure 4.

☒ Tick this box to confirm that a figure exemplifying the gating strategy is provided in the Supplementary Information.
