# Supplementary material for: TFPI from erythroblasts drives heme production in central macrophages promoting erythropoiesis in polycythemia
Source: Nat Commun. 2024 May 10;15:3976. doi: 10.1038/s41467-024-48328-8 (PMC11087540; doi:10.1038/s41467-024-48328-8)

Figure 1

Fig 1F

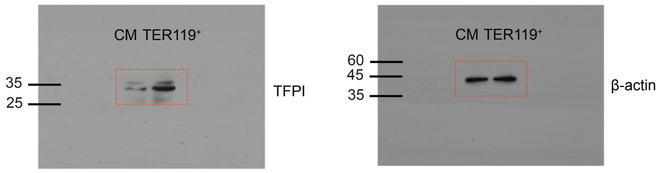

Figure 3

Fig 3J

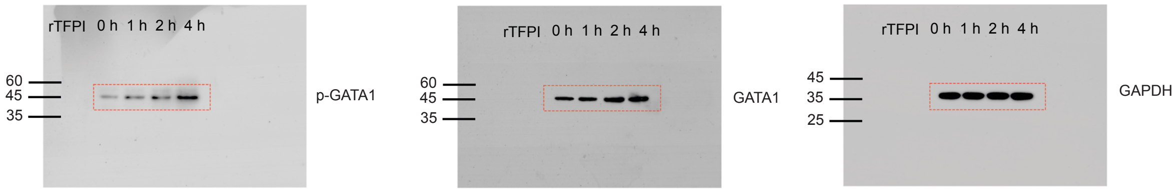

Fig 3K

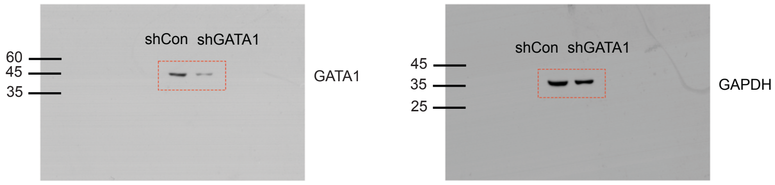

Figure 4

Fig 4F

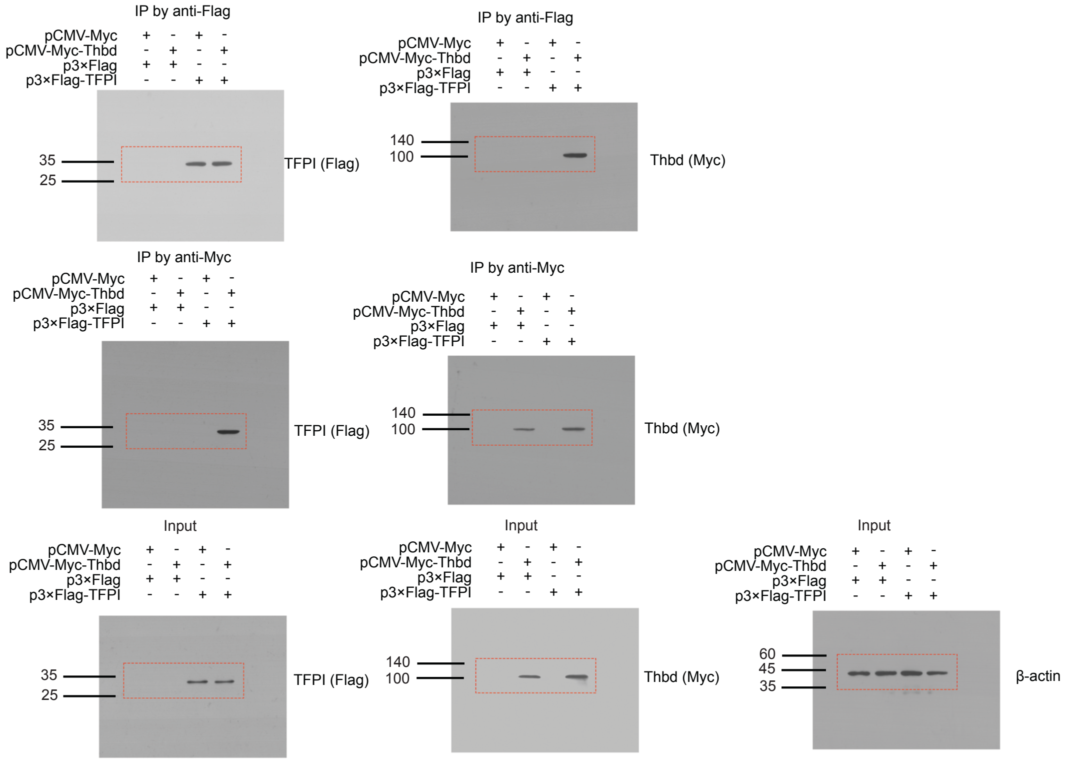

Fig 4H

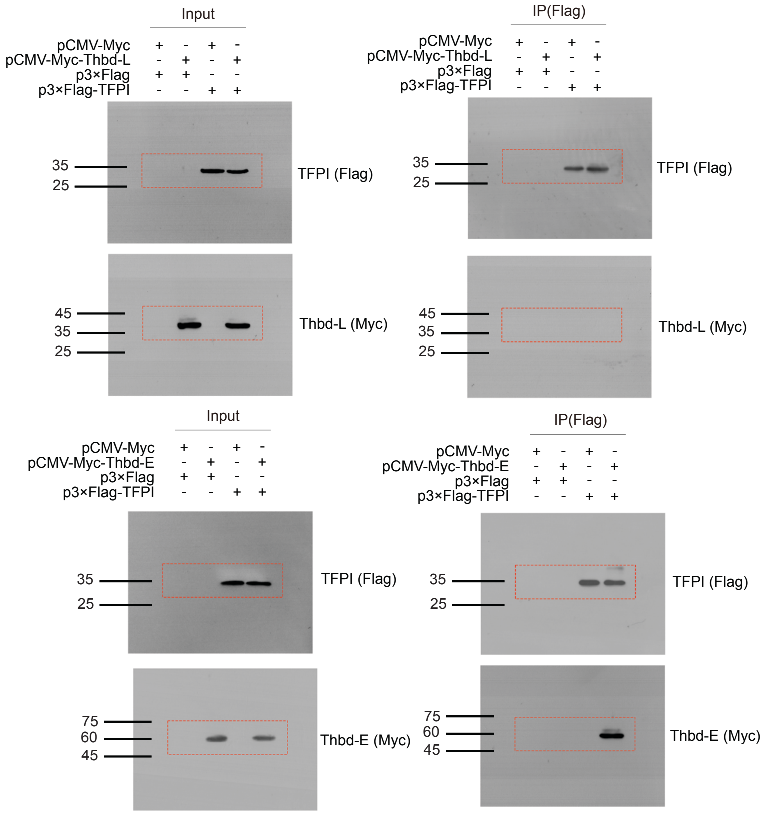

Fig 4I

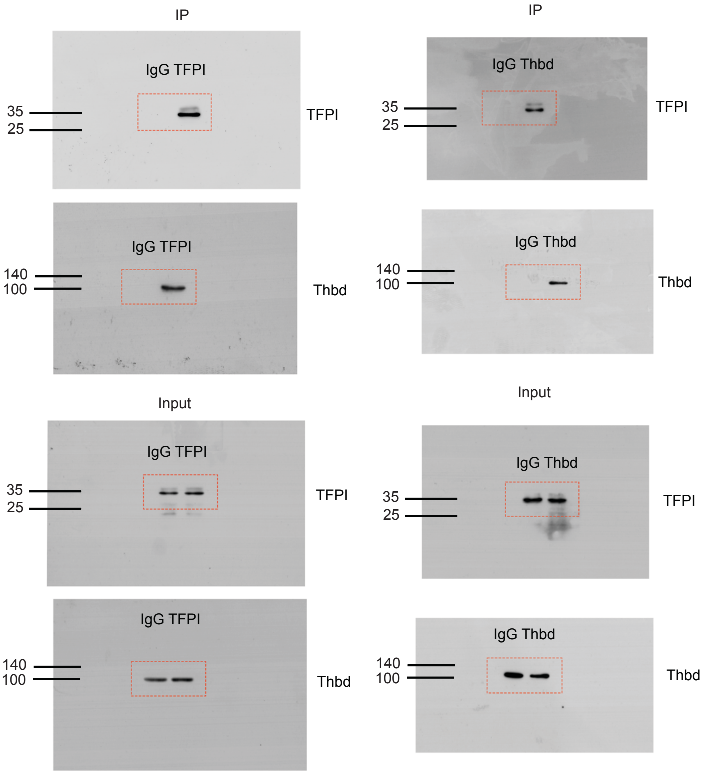

Fig 4J

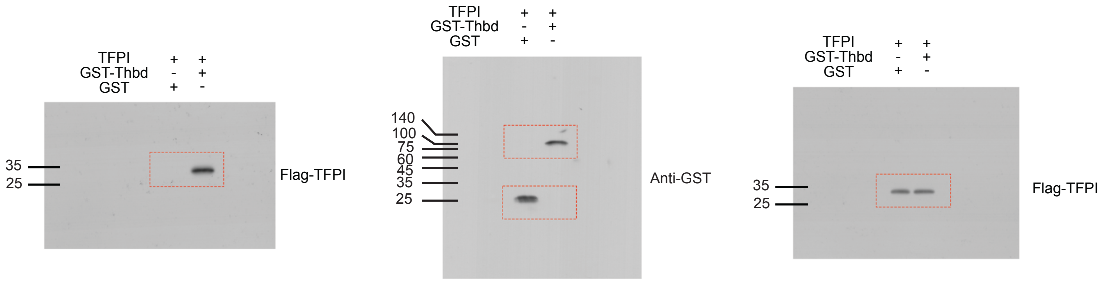

Fig 4K

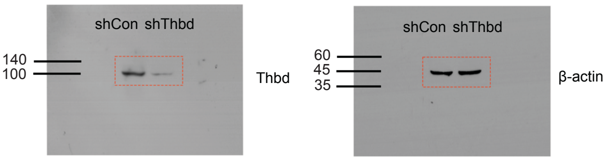

Figure 6

Fig 6B

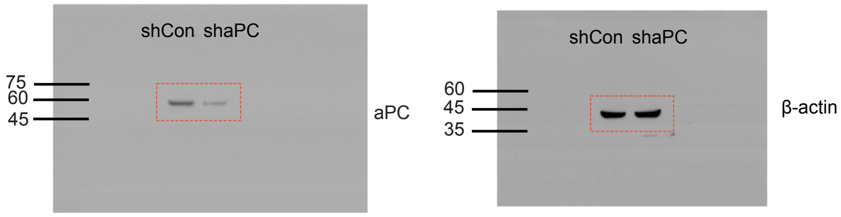

Fig 6G

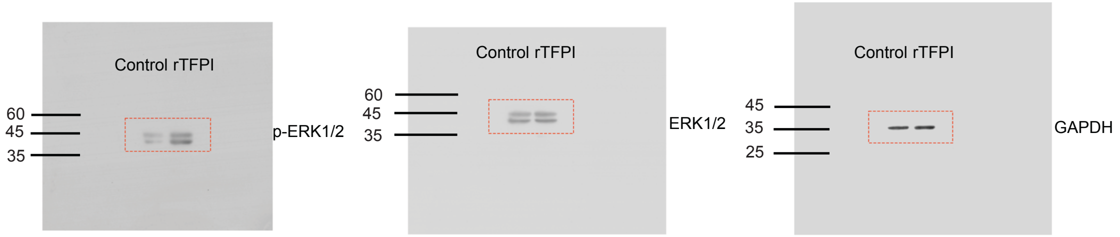

Fig 6H

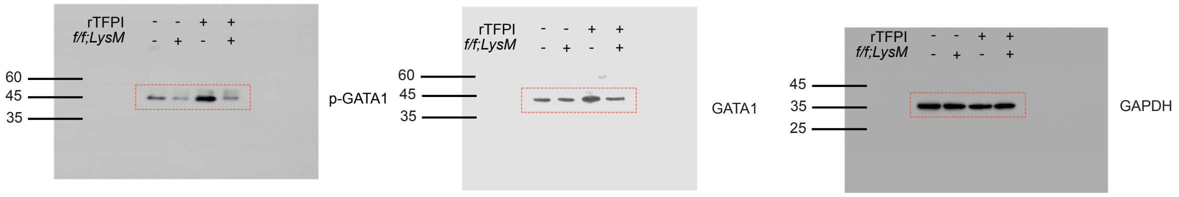

Fig 6I

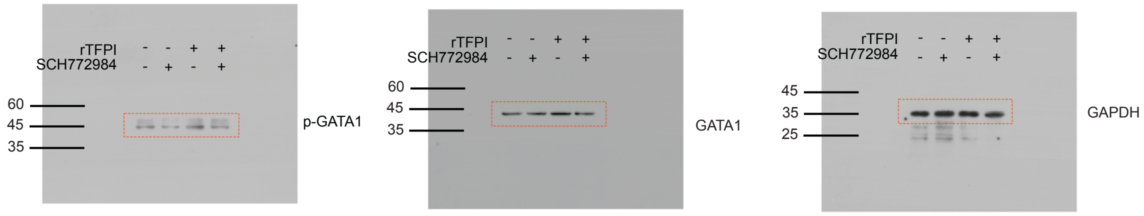

Fig 6J

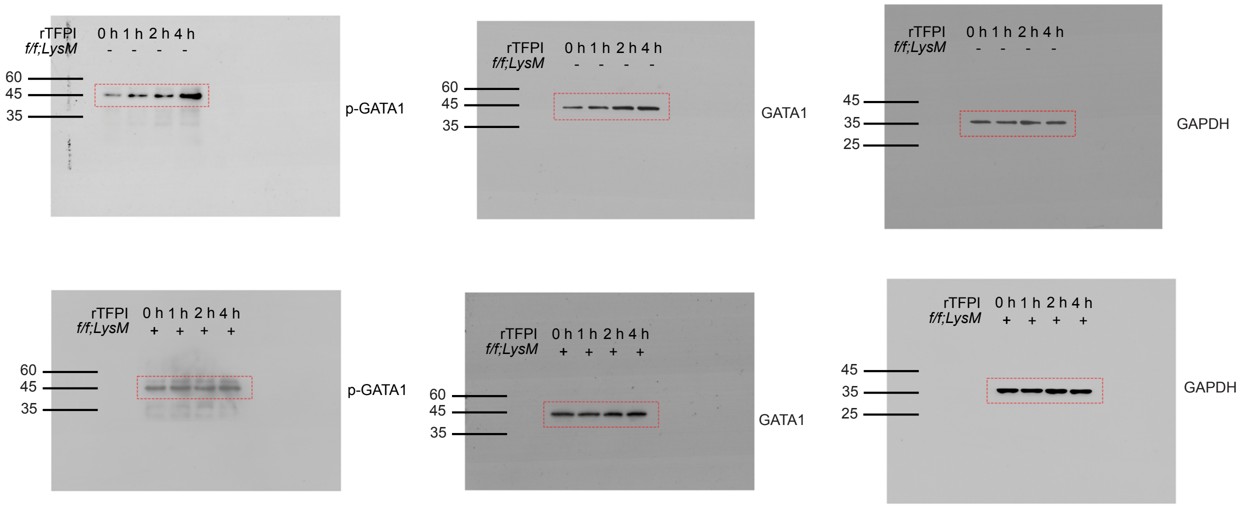

Figure 7

Fig 7B

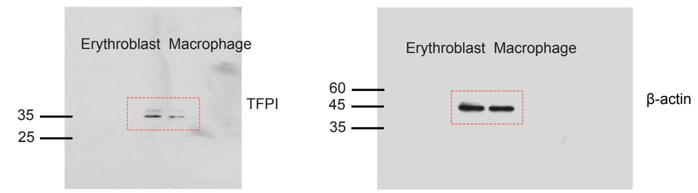

Fig 7C

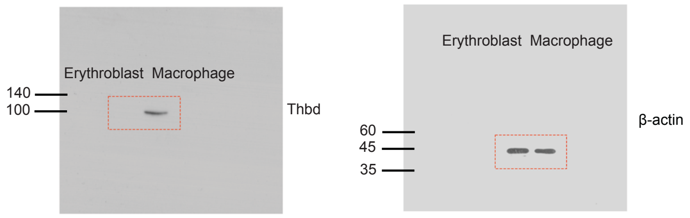

Figure S1

Fig S1B

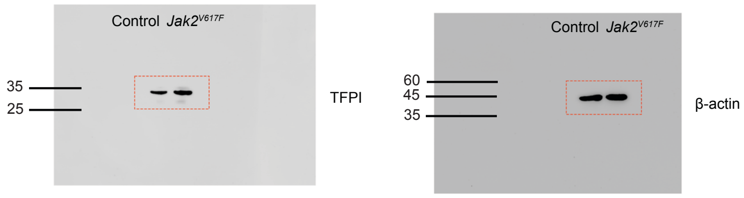

Fig S1C

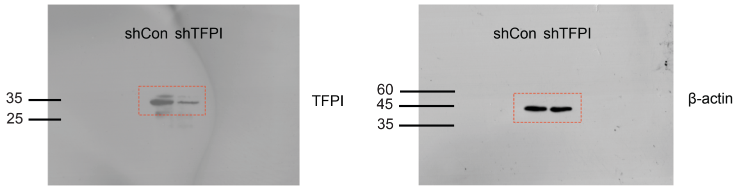

Fig S1J

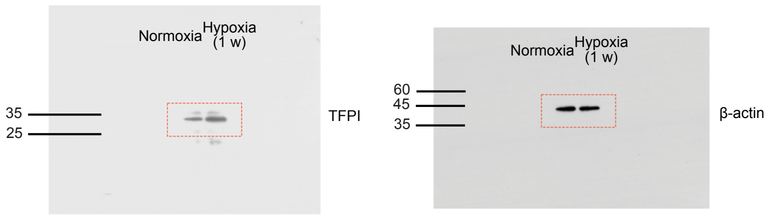

Figure S2

Fig S2B

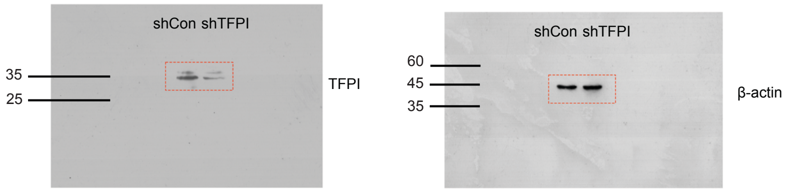

Figure S3

Fig S3B

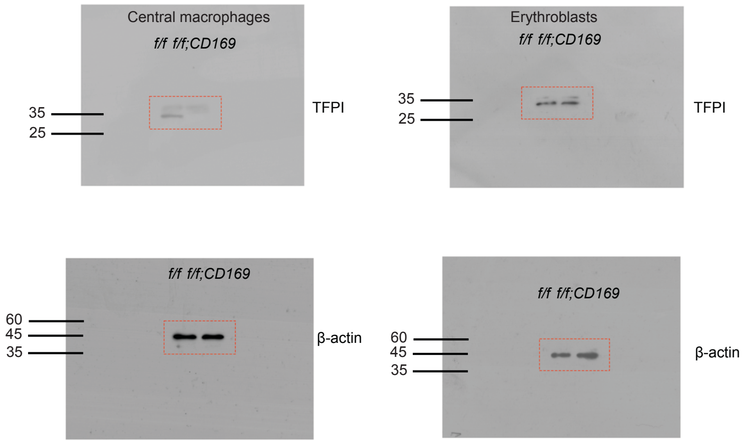

Figure S4

Fig S4B

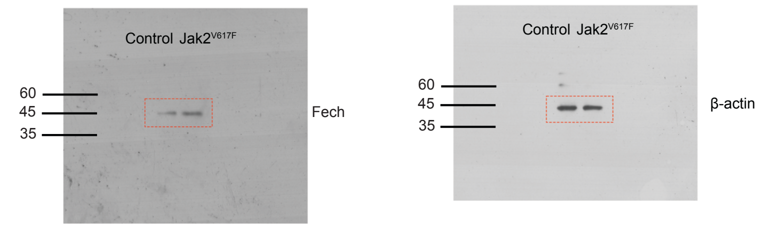

Fig S4C

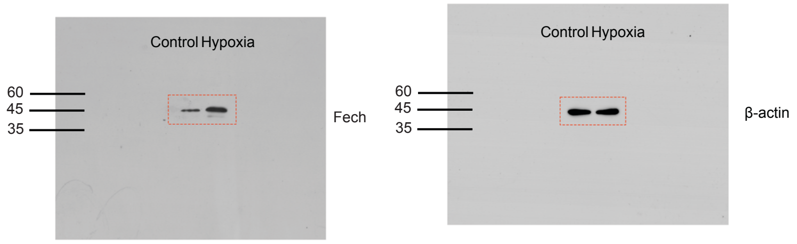

Fig S4D

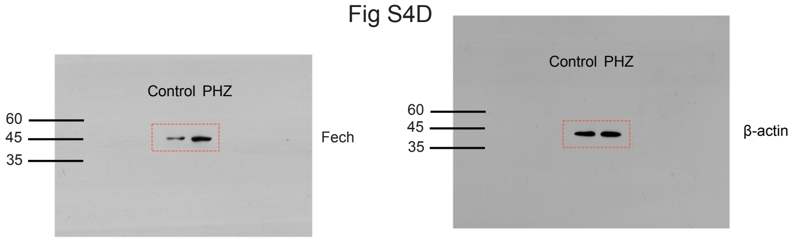

Figure S5

Fig S5C

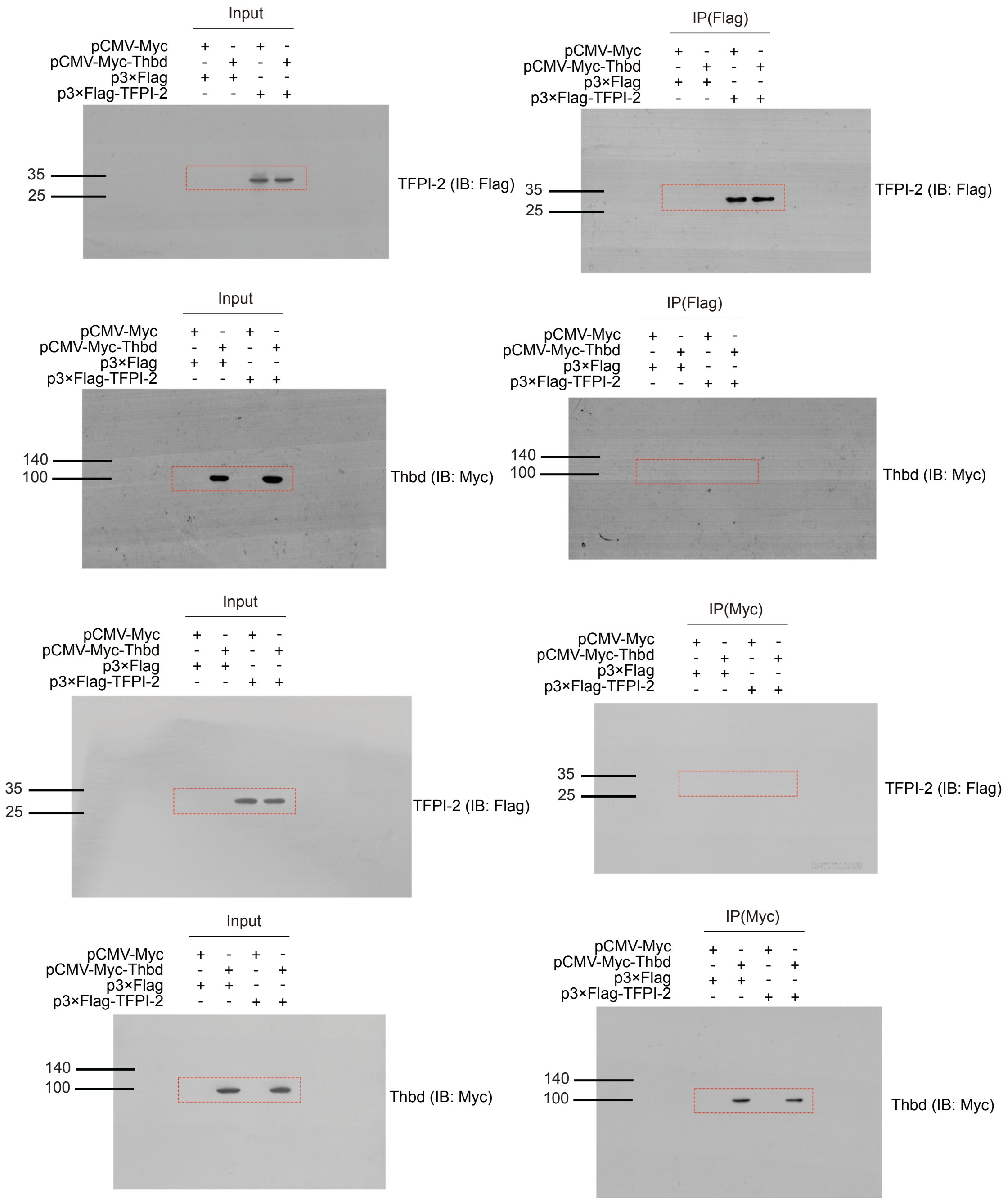

Supplement: Supplementary file 4 — Source Data [file 41467_2024_48328_MOESM4_ESM.zip › Uncropped Western blots.pdf]
